# Midwives’ practices on perineal protection and episiotomy decision-making: A qualitative and descriptive study

**DOI:** 10.18332/ejm/174126

**Published:** 2024-05-10

**Authors:** Silvia Rodrigues, Paulo Silva, Rosa Vieira, Ana Duarte, Ramon Escuriet

**Affiliations:** 1 Biomedical Sciences Institute Abel Salazar, Porto, Portugal; 2 Hospital of Braga, Braga, Portugal; 3 Health and Integrated Care division, Catalan Health Service, Barcelona, Spain

**Keywords:** labor stage, second phase, episiotomy, midwives, perineum

## Abstract

**INTRODUCTION:**

Perineal trauma is associated with both short- and long-term morbidity which in turn relates to the degree of trauma. The objective of this study was to understand midwives' practices regarding perineal protection during the second phase of labor, emphasizing decision-making to perform an episiotomy.

**METHODS:**

A descriptive and explanatory study was conducted with an intentional sample of twenty-two midwives working in the labor ward of a tertiary hospital in a metropolitan location and in the public service, in Portugal. A semi-open interview was applied to collect the data from 5 to 15 January 2019. The computer software package, NVivo version 10, was used to perform the thematic analysis.

**RESULTS:**

Four main themes arose from the midwives’ data: 1) Factors affecting the application of perineal protection techniques’, 2) Birth position, 3) Techniques for perineal protection, and 4) Episiotomy. The reasons for performing an episiotomy were the presence of tense perineum, large weight baby, previous obstetric anal sphincter injury, and Kristeller maneuver.

**CONCLUSIONS:**

Midwives' practices regarding perineal protection techniques and reasons for performing an episiotomy were not all in line with the evidence. Perineal massage was not mentioned as a perineal protection technique.

## INTRODUCTION

Perineal trauma occurs in approximately 85% of vaginal births^1^ and is associated with complications which, in turn, are related to the degree of trauma^2^. Women with an episiotomy or obstetric anal sphincter injury (OASIS) reported a worse birth experience than those with first- and second-degree perineal tears^3^. The World Health Organization does not recommend routine episiotomy for women giving birth vaginally^4^. According to a study carried out by Teixeira et al.^5^ there was a decreasing trend of OASIS in women with non-instrumented vaginal births and without episiotomy; from 2009, the OASIS rate decreased to 6.6 per 1000 in 2015 and the episiotomy rate also fell to 54% in 2015. However, in women with non-instrumental vaginal births with episiotomy, the rate of OASIS continued to increase reaching 3.9 per 1000 in 2015.

Wide variation in OASIS rates and in episiotomy rates is evident across countries. In data from 2012–2017, Canada, Denmark and United Kingdom had the highest rates of OASIS (2.5–3.1%) for spontaneous vaginal births^6^. However, Denmark had the lowest episiotomy rate (4.9%) in 2010 and Canada (17%) in 2007^7^. Portugal, Latvia and Poland had the lowest rates of OASIS (0.2–0.4%) for spontaneous vaginal births^6^. In contrast, Portugal and Poland had the highest episiotomy rate (68.2–72.9%) in 2010, but Latvia had 19.8% in the same year^7^. Converging results were provided in a recent Portuguese cross-sectional study in a birth center. The cross-sectional study found that the episiotomy rate for all spontaneous vaginal births was 47.4%, and 32.6% was performed on nulliparous women (n =570/1748) and the OASIS rate was 0.5%^8^.

This wide variation between countries in the same continent was explained, in part, by different models of care, which included different awareness of perineal management during the second stage of labor and consequently different intrapartum interventions. The midwife-led model of care was associated with fewer episiotomies and more satisfaction for women than with other care models^9^. Additionally, the midwife-led care model is recognized for ongoing support during labor that increases spontaneous vaginal birth and shortens the duration of labor. Furthermore, it reduces the use of any analgesia, negative feelings about birth experiences and a low Apgar score at five minutes^10^. According to a new approach in clinical practice that consists of the assistance of two midwives in the second stage of labor, it has been shown to reduce OASIS^11^. In contrast, the model of care that includes unnecessary interventions such as active pushing during the crowning of the fetal head, supine maternal position, acceleration with oxytocin for more than 30 minutes in the second stage of labor, and longer duration of second stage of labor, were risk factors associated with OASIS^12,13^. Evidence showed that the OASIS detection rate increased significantly after a hands-on workshop^14^. However, there was a significant decrease in the incidence of OASIS in departments with formal prevention programs and those without formal prevention programs, even when they were adjusted for other OASIS risk factors, meaning that the rate decreased regardless of formal prevention program implementation^15^. Thus, the aim of the study was to understand the midwives’ practices regarding perineal protection during the second stage of labor, highlighting the reasons for performing an episiotomy. This study is part of a larger study carried out at the same birth center.

## METHODS

### Design

The present study applied qualitative methodology to characterize midwives’ practices during labor and birth, at a birth center in Portugal. It is a descriptive and explanatory study describing the experiences and their meaning for individuals, considering that the person forms a whole with their environment, therefore their reality can only be understood within their context^16^.

The information collection technique was semi-open interview carried out individually by the main researcher of the study between 5 January and 15 January 2019. A total of twenty-two midwives voluntarily participated in the study and none withdrew. Participants before each interview gave their written consent. Each participant received a code that guaranteed their anonymity (P 1–22). According to the Data Protection Directorate, the data from the interview tapes and transcripts were stored securely in a locked folder in a closed office.

### Setting

The study setting included a birth center in northern Portugal. The birth center is within a tertiary Hospital, in a metropolitan location, and in the public service. The team is made up of 44 midwives and 30 obstetricians, handling 3000 births per year. The episiotomy rate for all spontaneous vaginal births was 47.4% and OASIS rate was 0.5%^8^. One-to-one care is not implemented in the hospital and depending on clinical routines, midwives may be responsible for several women simultaneously. If complications occur during labor, midwives work collaboratively with obstetricians.

### Participants recruitment

Sampling was intentional among midwives employed at a birth center in northern Portugal. The inclusion criterion was that participants must be employed at the birth center. The sample size was estimated according to another study in this field^17^. The head nurse explained the study objectives to the team and provided the midwives with the contact details of one of the researchers. Midwives expressed their interest in participating by contacting one of the research team members via cell phone.

### Measures and data collection

For data collection, the researcher contacted by interested participants carried out semi-open and face-to-face interviews. The researcher who conducted the interviews had previously carried out a qualitative study. The interviews took place at a time according to the availability of the participants, with only the presence of the interviewer and the participant, at the beginning of 2019 in an office at the birth center. An interview schedule provided to the authors of the Midwives Expertise Protection Perineal Intact (MEPPI) study published in 2017^17^, was used as guidance to ensure consistency between interviews. The interview schedule has nine questions and is divided into three topics: Awareness of perineal protection, clinical practice of midwives during the second stage of labor, and risk factors for perineal trauma. Participants responded in writing to sociodemographic questions (age and marital status), they also answered about how long they have been working as a midwife, how long they have been applying techniques and interventions to maintain an intact perineum and whether they received theoretical and practical classes on perineal protection techniques. Each set of responses was identified by the participant’s code number. All interviews were digitally recorded and later transcribed verbatim. The average duration of the interviews was 26.1 minutes (SD=6.0; range:15–36).

### Analysis

The transcribed data from each interview were presented to each participant for validation. The computer software package, NVivo version 10, was used to perform the thematic analysis. The initial results of the first five interviews were discussed by the authors. Then, the initial results were compared with new data from the following interviews, in order to look for similarities and disparities, based on the constant comparative method. When participants presented opposing points of view, in the following interviews we questioned the participants about the topic. The data resulting from the interview transcriptions were exchanged between the two main researchers and subsequently analyzed, compared, and reached a consensus. The final analysis was tested by ‘peer debriefing’. The other authors analyzed 22 transcripts of participant interviews and draft results. When presenting the results, we used the midwives’ own words to ensure the accuracy of the analysis.

## RESULTS

### Participant characteristics

Twenty-two midwives participate in the study. The period of time working as a midwife was 14.1 years (SD=10.0), range 1–32 years. Twelve of them (54.5%) received theoretical and practical classes on perineal protection techniques. Table 1 summarizes the participant characteristics.

**Table 1:** Characteristics of participating midwives of a birth center in Braga, Portugal, 2019 (N=22)

*Characteristics*	*Mean (SD), range*
Period of time working as a registered midwife (years)	14.1 (10.0), (1–32)
Period of time using techniques and interventions to maintain the perineum intact (years)	8.1 (5.1), (1–15)
Theoretical and practical classes on perineal protection techniques, n (%)	12 (54.5)

Source: Research Data, 2019.

Four main themes emerged from the analysis of midwives’ data on practices at a birth center in Portugal. These were: 1) Factors affecting the application of perineal protection techniques, 2) Birth position, 3) Techniques for perineal protection, and 4) Episiotomy. Table 2 lists the codes, the number of participants who referred to the code, the frequency with which the code was used by participants, and the links between the codes and the categories and themes. The four themes identified were not mentioned in equal proportion. The theme most developed by the midwives was ‘Birth position’.

**Table 2:** Themes, categories and codes of the birth center in Braga, Portugal, 2019 (N=22)

*Themes*	*Categories*	*Code*	*Number of participants who used the code*	*Number of times code used*
**1. Factors affecting the application of perineal protection techniques**	Lack of continuous presence of the midwife	One-to-one	16	28
**2. Birth position**	Alternative birth position	Comfortable birth position to woman	18	39
	Avoid lithotomy	Free movement of sacrum	17	25
		Upright position	6	12
**3. Techniques for perineal protection in second stage of labor**	Warm compresses	Apply warm compresses	10	20
	Hands-on	Controlled head delivery	12	24
		Flexing the head delivery	10	10
	Hands-off	Don’t touch	8	12
	Spontaneous pushing	Let the women push	16	40
**4. Episiotomy**	Reasons to perform an episiotomy	Tense perineum	18	26
		Large baby (weight)	10	15
		Previous obstetric anal sphincter injury	8	12
		Kristeller maneuver	12	16

Source: Research Data, 2019.

### Theme 1: Factors affecting the application of perineal protection techniques

Sixteen midwives, with a mean age of 41.5 years (SD=10.5; range: 27–60), average period of time using techniques and interventions to maintain the perineum intact 7.5 years (SD=5.4; range: 1–15) discussed the importance of midwife’s continuous presence during labor. Ten of them (45.5%) stated that when they have the opportunity to provide continuous supportive care, they do not need to examine the woman so often. This theme showed that midwives think that the lack of continuous presence with women in labor maybe be a factor affecting the application of perineal protection techniques:

*‘When I am one to one woman, in early second stage, the upright position is good to improve the progression of the baby and she feels more safe and supported, but most of the times I´m not one to one.’* (P16)

*‘I feel my continuous presence and positive feedback make difference in the perineum outcomes and I don’t need to touch so many times.’* (P22)

*‘When I arrive to do the birth, and I never touched the woman, of course I have doubts about the perineum.’* (P 5)

*‘I can’t always be one to one, I feel something is missing, the protection of the perineum can be a missed care.’* (P1)

### Theme 2: Birth position

An alternative birth position to lithotomy was suggested by the seventeen (68.2%) midwives interviewed as a measure to protect the perineum. The average age of the midwives was 44.9 (SD=11.2), ranging 27 to 60 years, the average time of using techniques and interventions to maintain the perineum intact was 8.2 (SD=5.5), range from 1 to 15 years. Two categories dominated the theme “birth position”. These were “Alternative birth position” and “Avoid lithotomy”. Participants expressed their clinical practice regarding the maternal birth position during the second stage of labor.

### Alternative birth position

*‘I encourage the woman to adopt the most comfortable birth position to her.’* (P13)

*‘The woman is free to push in the birth position that she wanted, and I allowed and encourage it.’* (P19)

*‘Usually, I suggest the sitting upright position as the one of the more comfortable.’* (P21)

*‘I think the most protective is the lateral birth position.’* (P13)

### Avoid lithotomy

*‘To protect the perineum I avoid the lithotomy birth position, only if the woman wants.'* (P1)

*‘I avoid the lithotomy position because the woman doesn’t have free movement of the sacrum.’* (P6)

### Theme 3: Techniques for perineal protection in the second stage of labor

All the midwives talked about the hands-on technique to protect the perineum during birth. Others said that they use warm compresses. Eight (36.4%) midwives stated that they sometimes prefer to use the hands-off technique. The average age of midwives was 39.8 years (SD=11.4; range: 27–57), the average time of using techniques and interventions to maintain the perineum intact was 6.0 years (SD=5.5; range:1–15). There were four categories under the theme. These were ‘Warm water’, ‘Hands-on’, ‘Hands-off’ and ‘Spontaneous pushing’. Participants indicated how they protect the perineum during the second stage of labor:

Comments regarding ‘Warm water’ were:

*‘To protect the perineum I apply warm compresses.’* (P22)

*‘My favorite technique is warm compresses in the perineum during the second stage of labor.’* (P3)

for ‘Hands-on’:

*‘To protect the perineum, I hold the perineum with right hand and hold the head with my left hand. Sometimes I flexing the head.’* (P16)

and ‘Hands-off”:

‘Do nothing, I don’t touch in the perineum.’ (P8)

‘During the second stage of labor, I try to touch as less as possible, or never.’ (P14)

while for ‘Spontaneous pushing’, most midwives talked about how they helped the woman maintain control during the active second stage:

*‘I leave the woman push by herself, spontaneously, I wait for her spontaneous push.’* (P1)

*‘Sometimes, when there is not an effective push, I need to encourage the woman for an active and directed pushing.’* (P17)

### Theme 4: Episiotomy

All the midwives agreed to performed an episiotomy when there were signs of fetal distress. However, they included more reasons to perform an episiotomy. The reasons were: a women with a previous obstetric anal sphincter injury, when the head is crowing and the perineum is bleeding, if someone performs a Kristeller maneuver, baby large, and when there are signs of a tense perineum. Participants expressed concern about preventing OASIS and at the same time preventing neonatal morbidity. The theme includes the category ‘Reasons to perform an Episiotomy’, with comments:

*‘When the woman had a previous obstetric anal sphincter injury, I prefer to do an episiotomy, since she could have more risk to have other severe laceration again.’* (P 9)

*‘During the second stage of labor, when the head is crowning and the perineum start bleeding I prefer to do an episiotomy.’* (P21)

*‘If someone performs a Kristeller maneuver I do an episiotomy.’* (P17)

*‘When the baby could be large I do an episiotomy.’* (P 6)

*‘Sometimes when the woman has a tense perineum, I need to perform an episiotomy.’* (P 4)

*‘Birth performed by a midwife does not need an episiotomy.’* (P11)

## DISCUSSION

The four themes identified were: 1) ‘Factors affecting the application of perineal protection techniques, 2) ‘Birth position’, 3) ‘Techniques for perineal protection in second stage of labor’, and 4) ‘Episiotomy’.

Theme 1 showed that midwives needed one-to-one care to promote perineal integrity. This is in line with the low rates of episiotomy found in the MEPPI study, with midwives employed in midwife-led units in Ireland and midwives working in home and community in New Zeland^1^. Midwife-led units have the model of care recognized by one midwife to one woman in labor. In Portugal, the usual care model generally implemented during labor is characterized by more than one laboring woman for one midwife and midwives often have simultaneous responsibilities. A systematic review about continuous support care during labor found that considerable time is spent managing technology, records, and ensuring adherence to institutional protocols by nurses and midwives. Furthermore, work shifts that begin or end in the middle of a woman’s labor compromise the continuous support care^10^. The significant impact of missed care on patient quality and safety outcomes was described in a scoping review^18^. In addition, the phenomenon can be extended to different areas of nursing care^18^. Another study added that midwives’ decision making was influenced by the model of care, the complicated environment and power relations between clinicians and midwives^19^. In the present study, the midwives did not mention the complicated environment or problems with obstetricians as a factor affecting the application of perineal protection techniques.

Theme 2 was the most developed by the midwives. According to these midwives, they encourage women to choose the position they feel most comfortable during labor and birth. In recent evidence, there has been an increase in perineal trauma in the horizontal birth position^20^, fetal heart rate abnormalities and fewer spontaneous vaginal births than in the upright or side-lying position^21^. According to MEPPI study, midwives favored the ‘all-fours’ birth position, as it favors observation of the perineum, decreases pressure and consequently less perineal trauma^1^. A systematic review reported a reduction in the rate of episiotomy and OASIS in free sacrum birth position, and there was an increase in the rate of minor trauma^22^. Despite the evidence, one study demonstrated that obstetricians and midwives continue to assist women in the horizontal birth position in accordance with their routine and cultural norms^23^. In a cross-sectional study carried out at the same birth center, with a sample of 1748 spontaneous vaginal births, only 355 (20.3%) were in an alternative birth position^8^. Therefore, obstetricians and midwives need more opportunities to develop skills or not lose skills to support women in the upright birth position^23^. In the present study, the average number of years that midwives have worked as registered midwives was 14.1 years (SD=10.0), although the average number of years using the techniques and interventions to preserve the perineum integrity was 8.1 years (SD=5.1). However, only twelve (54.5%) midwives received theoretical and practical classes on perineal protection techniques. Another study suggested that midwives’ knowledge and beliefs are developed through their formal education, and professional and personal experiences^19^. Midwives need formal education and professional experience on evidence-based techniques and interventions to protect the perineum. Therefore, theoretical and practical classes regarding flexible sacrum position should be taught to midwifery students and midwives. Furthermore, pregnant women should be taught and trained during pregnancy regarding the flexible sacrum position, in order to promote women’s autonomy in choosing the birth position. This may increase the number of women adopting flexible sacrum positions in hospital settings^24^.

Theme 3, the midwives reported the hands-on technique as the most used (hands-on technique include the controlled of head delivery and sometimes flexing the head during birth), followed by warm compresses and a smaller number said they used hands-off technique. Interventions to protect the perineum on the second stage of labor have been extensively studied and recommendations^25^ have been made. Perineal massage performed with water soluble-lubricant, using the index and middle fingers, to gently stretch the perineum^26^, increase blood flow and elasticity^27^. Moreover, a systematic review exclusively on the perineal massage technique during labor demonstrated, in nulliparous women, an increased incidence of perineal integrity and a decreased incidence of episiotomy. While, in multiparous women there was an increase in the incidence of perineal integrity^26^. In addition, a Cochrane systematic review reported a decrease in OASIS^2^. In our study, despite the evidence and recommendations^25^ about perineal massage during the second stage of labor, the midwives interviewed did not mention the perineal massage technique. On the contrary, some midwives reported applying the warm compresses technique and this is a recommended perineal protection technique^2,25^. In the present study the ‘Hands-on’ technique was the most applied, although the ‘Hands-off’ technique is recommended over the ‘Hands-on’ technique^25^. In addition, the ‘Hands-on’ technique should be replaced by the perineal massage technique. A recent discussion paper suggests that the ‘Hands-on’ technique interferes with the normal birth process to slow the fetal head exit. On the other hand, adequate communication with the woman (between or at the end of the contraction) can help the woman to slow the fetal head exit^28^. Regarding to push spontaneously, some midwives have encouraged women to push whenever they want. However, they highlighted that they gave instructions on how to push when the active second stage of labor was longer, the woman reported tiredness or was not effective. Furthermore, a recent biomechanical study^29^ suggested that maternal pushing should last no more than 5 seconds compared to each push lasting 10 seconds. The recommendation is that the women push according to their own pushing or using Valsalva maneuvers^25^. In the present study, the midwives’ practices regarding application of perineal protection techniques were not in accordance with the evidence.

Theme 4, the midwives mentioned that the main reason to perform an episiotomy was the presence of a tense perineum. According to Aquino et al.^26^, the perineal massage during the second stage of labor could support the midwives to reduce the episiotomy rate^26^. In the present study, some midwives reported performing an episiotomy when the women had a previous OASIS. The literature does not support a policy of routine episiotomy, because a systematic review demonstrated that in women expected to have a spontaneous vaginal birth, a policy of selective episiotomy can decrease OASIS by 30%^30^. Another reason mentioned by midwives was a large weight baby. Although, in the MEPPY study, midwives did not consider that a large-weight baby can be a major influence on perineal trauma^17^. Likewise, in our study, few midwives mentioned that the Kristeller maneuver was a reason to perform an episiotomy. The Kristeller maneuver is not reported or is underreported in studies about this field. According to Youssef et al.^31^, the Kristeller maneuver was associated with more than 2-fold risk of levator ani avulsion, after adjusting for potential confounders such as maternal age, body mass index, length of first and second stage of labor, episiotomy, epidural analgesia and newborn weight^31^. Consequently, injury to the levator ani muscle is associated with a significantly increased risk of developing pelvic floor dysfunction, especially pelvic organ prolapse^32^. In the literature, there are no published articles that support the reason for performing an episiotomy when applying the Kristeller maneuver. In addition, the Kristeller maneuver is not recommended during the second stage of labor^4^. In our study, only one midwife stated that she did not perform episiotomies. However, one study demonstrated that there is a negative association between very low rate of episiotomies and OASIS^33^. Despite this, one study suggested that the episiotomy rate could decrease because the increase of OASIS rate appears to be a better diagnosis of OASIS rather than the episiotomy rate^5^.

According to another study^34^ on the implementation of best practices during labor, it is suggested to create midwife platforms to discuss issues regarding clinical practice in the labor ward. Additionally, workshops were suggested to disseminate recent evidence on intrapartum care and develop skills. A newsletter could be developed by the team to disseminate the team’s findings on women’s results. These results could be shared with other birth centers and with women. This set of activities aims to keep the team engaged and thus be able to translate the evidence into practice.

### Strengths and limitations

This study has several strengths, these include that all the midwives interviewed were motivated to participate and talk about the topic, the fact that the high rate of episiotomy in the birth center is also found across other countries, and the use of an interview schedule provided for the MEPPI study published in 2017^16^. However, the study has the limitation of having been carried out in one birth center in only one country, and the results are not applicable to other birth centers.

## CONCLUSIONS

The results of this study allow us to understand the midwives’ practices regarding perineal protection during the second stage of labor and the reasons for performing an episiotomy. Four topics emerged from the analysis: 1) ‘Factors affecting the application of perineal protection techniques’, 2) ‘Birth position’, 3) ‘Techniques for perineal protection’, and 4) ‘Episiotomy’. Continuous care was indicated as a factor affecting the application of perineal protection techniques. Perineal massage was not mentioned as a perineal protection technique. Midwives reported the importance of promoting free movement of the sacrum and avoiding a horizontal birth position, but encouraged women to adopt the birth position they prefer. The most described perineal protection techniques were ‘Hands-on’, followed by warm compresses and at last by ‘Hands-off’. Finally, the midwives described that perineum tense, previous OASIS, large-weight baby and Kristeller maneuver were reasons for performing an episiotomy. Midwives’ practices regarding perineal protection techniques and reasons for performing an episiotomy were not all in line with the evidence. Therefore, theoretical and practical classes regarding perineal protection techniques during the second stage of labor and reasons to perform an episiotomy should be taught to midwifery students and midwives. Furthermore, the pregnant women should be taught and trained during pregnancy regarding perineal protection techniques during the second stage of labor, in order to promote women’s autonomy in choosing the perineal protection technique during the second stage of labor.

## Data Availability

The data supporting this research are available from the authors on reasonable request.
